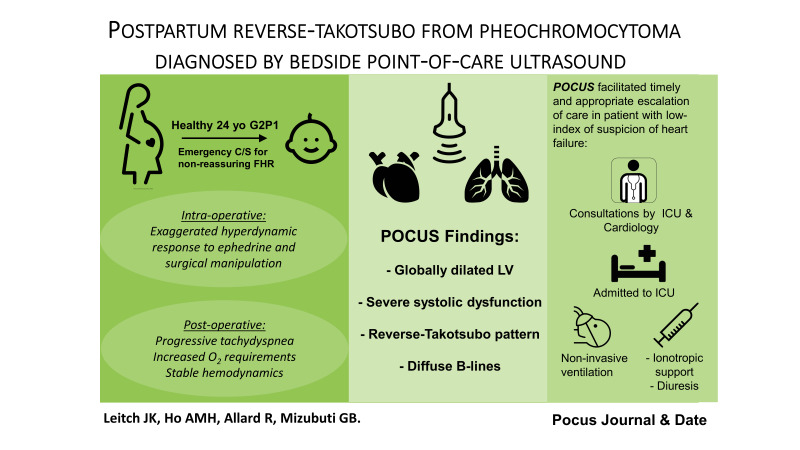# Postpartum reverse-Takotsubo from pheochromocytoma diagnosed by bedside point-of-care ultrasound: A case report

**DOI:** 10.24908/pocus.v5i2.14432

**Published:** 2020-11-18

**Authors:** Jordan K Leitch, Anthony M.-H Ho, Rene Allard, Glenio B Mizubuti

**Affiliations:** 1 Department of Anesthesiology and Perioperative Medicine, Kingston Health Sciences Centre, Queen's University Kingston, ON Canada

**Keywords:** Point-of-Care Ultrasound, Pregnancy, Cardiomyopathy, Pheochromocytoma, Cardiogenic shock

## Abstract

Point-of-care ultrasound is invaluable in the setting of obstetric anesthesia, where the differential diagnosis for dyspnea, hypoxemia and/or hemodynamic abnormalities is broad. This report describes a previously apparently healthy parturient with an uncomplicated pregnancy at 35-weeks gestation who underwent an emergency cesarean section under general anesthesia due to severe acute abdominal pain and fetal bradycardia. Intraoperatively, she presented with severe hypertension and tachycardia that were difficult to control and associated with ischemic ECG changes. In the immediate postoperative period, she developed retrosternal tightness and dyspnea, and a bedside point-of-care ultrasound scan revealed a grossly dilated and hypokinetic left ventricle, as well as diffuse B-lines throughout all lung fields – consistent with cardiogenic pulmonary edema. She was admitted to the intensive care unit, where she recovered over several days. Pheochromocytoma was subsequently diagnosed, and she eventually underwent uneventful elective adrenalectomy after appropriate endocrine and hemodynamic optimization.

## Introduction

The use of point-of-care ultrasound (POCUS) in acute care medicine has grown exponentially. Given its non-invasiveness, portability, and excellent image resolution, ultrasound (US) machines allow clinicians to quickly – and serially – assess an unstable patient at the bedside, and guide management by ruling in or out important differential diagnoses which often present conflicting management goals. POCUS is invaluable in the setting of obstetric anesthesia where the differential diagnosis for dyspnea, hypoxemia and/or hemodynamic abnormalities is particularly broad – and often life-threatening 1. 

We present a case in which a previously (apparently) healthy parturient with an uncomplicated pregnancy presented antepartum in a hyperdynamic circulatory state which worsened during emergency cesarean section. When she developed mild dyspnea (SpO_2 _= 95% on room air) post-partum, a POCUS examination revealed a severely hypokinetic and dilated left ventricle, which triggered a cardiology consultation and critical care admission. As such, POCUS facilitated the rapid diagnosis of congestive heart failure and timely management with non-invasive ventilation, inotropic support and diuresis in a patient in whom cardiogenic shock would have otherwise been substantially further down the diagnostic pathway. Furthermore, prompt identification of severe cardiomyopathy (in addition to the patient’s intra-operative hemodynamic state) provided the impetus to recommend imaging and urine metanephrine testing, which confirmed the diagnosis of pheochromocytoma, and allowed for its appropriate management. Notably, these investigations were critical to distinguishing pheochromocytoma-induced cardiomyopathy from peripartum cardiomyopathy, the latter of which poses a significant risk to future pregnancies (recurrence rates: 25-100% 2). Local ethics approval and written consent were obtained for publication of this report.

## Case Description

A 24-year-old parturient G2P1 (35 weeks+2 days) was admitted with a 3-hour history of acute and rapidly progressive lower abdominal pain and non-reassuring fetal heart rate (FHR) not associated with vaginal bleeding/discharge. The patient reported an otherwise uncomplicated pregnancy and denied substance abuse. Her past medical history was unremarkable. On arrival, her blood pressure (BP) was 135/82 mmHg, heart rate (HR) 70 bpm, SpO_2 _= 98%, and FHR 115 bpm. Fetal movement was reportedly absent since the onset of pain. Cervical examination revealed intact membranes and no bleeding/discharge. Uterine irritability was obvious on abdominal examination, with no evidence of peritoneal irritation. Bedside US revealed an anterior placenta with no signs of abruption, and an infant in cephalic presentation with now sustained bradycardia (85 bpm). The patient was immediately transferred to the operating room (OR) due to suspicion of uterine rupture and/or placental abruption. 

In the OR, ECG, SpO_2_, and non-invasive BP were applied, and general anesthesia (GA) was induced, followed by tracheal intubation. Anesthesia was maintained with sevoflurane (Et_SEVO_~2%). Given the possibility of ongoing hypovolemia, crystalloids were rapidly infused through 2 peripheral 18G intravenous lines and ephedrine 10 mg was administered upon induction. This triggered an unusual and disproportionate hypertensive (215/110 mmHg) response associated with ECG changes (ST depression and T wave inversion) which was managed with propofol (50 mg). A Pfannenstiel incision was performed and the infant delivered without complication. Given persistent hypertension (systolic BP 170-200 mmHg) and tachycardia (130 bpm), incremental doses of fentanyl and morphine (total of 250 mcg and 10 mg, respectively) were administered on the assumption that the hemodynamics were pain-related, with negligible effect. No free fluid/blood was noted upon entry into the abdomen, and the uterus appeared normal (no evidence of Couvelaire uterus). Amniotic fluid was clear, and the placenta did not show evidence of abruption. The placenta appeared smaller than normal consistent with chronic ischemia, which also raised the possibility of acute uterine ischemia as a cause of her abdominal pain. Urinary bladder, ovaries and fallopian tubes were intact. Given the lack of an obvious reason to explain the patient’s acute and severe preoperative abdominal pain, general surgery was consulted intraoperatively; however, no intra-abdominal pathology was identified. Notably, intraoperative palpation of the patient’s right upper abdominal quadrant triggered severe hypertension (220/100 mmHg) and tachycardia (135 bpm). This was treated with hydralazine (40 mg), and prompted the anesthesia team to arrange for phentolamine as undiagnosed pheochromocytoma was now suspected. Estimated blood loss was 500-700 mL, and the patient received a 1500 mL of crystalloid. Upon completion of surgery and tracheal extubation, the patient was transferred to the post-anesthetic care unit in stable condition (BP = 142/90 mmHg, HR = 120 bpm, SpO_2 _= 97% on room air). 

**Figure 1:**
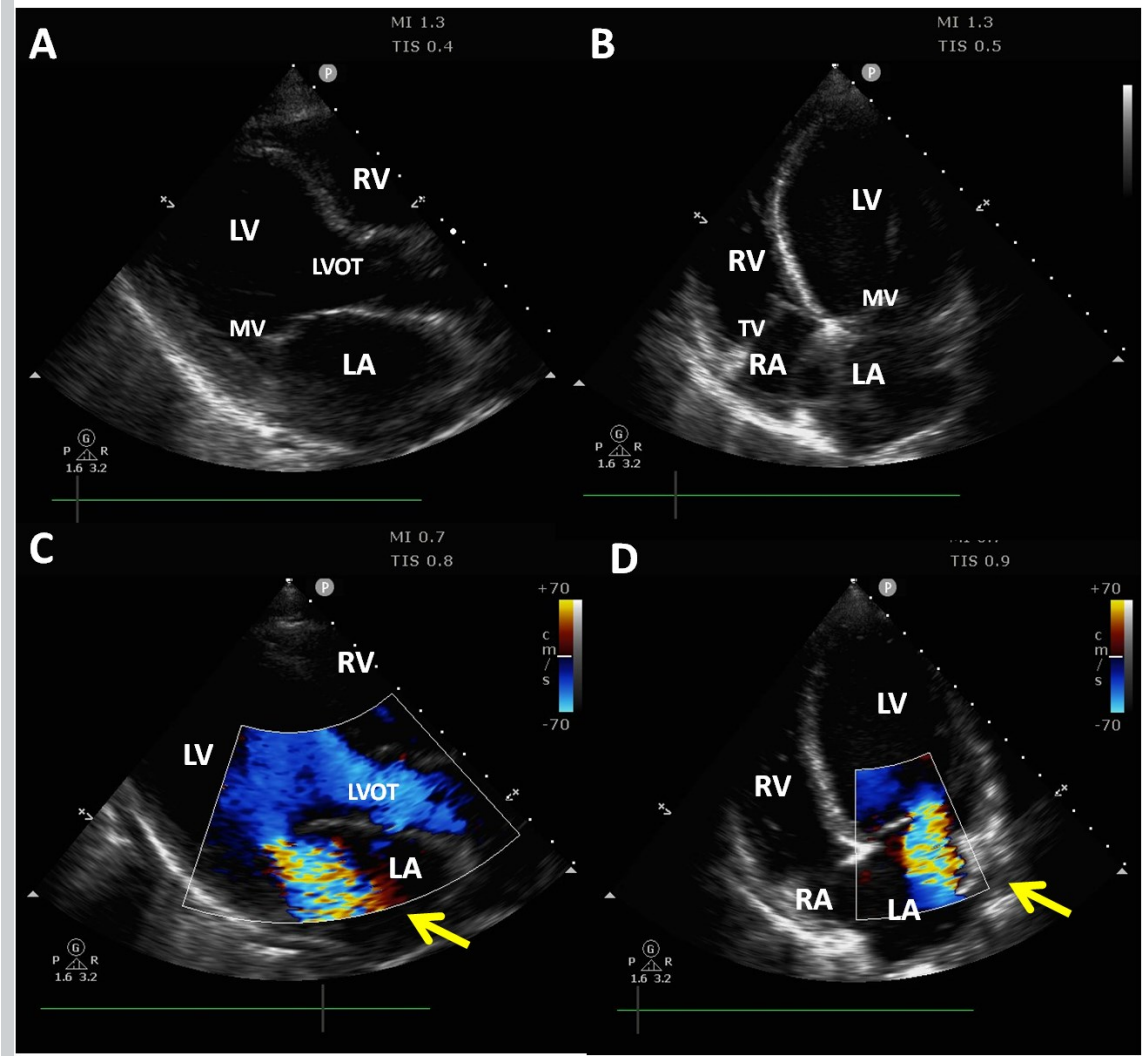
Parasternallong axis (A) and apical 4-chamber (B) views showing a dilated left ventricle(LV). Similar views are shown in panels (C) and (D), respectively, with colorDoppler demonstrating significant mitral regurgitation (yellow arrow). RV =Right ventricle; LVOT = Left ventricular outflow track; RA = Right atrium; MV =Mitral valve; TV = Tricuspid valve.

Shortly after, the patient complained of progressive retrosternal tightness and shortness of breath. She was tachypneic (26 rpm) and diaphoretic, with bilateral basal crackles. Vital signs were BP = 145/89 mmHg, HR = 110 bpm, and SpO_2 _= 95% on oxygen (15 L/min) through a non-rebreather facial mask. Blood work revealed an elevated CK (262 U/L) and troponin (1111 ng/L). Toxicology profile was negative and the remaining blood tests (CBC, electrolytes, preeclampsia panel, TSH and PTT/INR) were within normal range. A bedside POCUS revealed a globally dilated left ventricle with severe systolic dysfunction and wall motion abnormalities consistent with reverse-Takotsubo pattern (basal and mid-segment hypokinesis/akinesis and apical segment hyperkinesis), normal right ventricular function, no pericardial effusion, severe mitral regurgitation, and mild-to-moderate tricuspid regurgitation (Figure 1, online Videos S1-5). These findings were readily apparent to the operators (JKL and GBM) and later confirmed by a formal transthoracic echocardiogram (without contrast) performed by a certified echocardiographer. Bilateral multiple B-lines were identified on lung US. At this point, BiPAP, dobutamine infusion and furosemide 40 mg were administered upon transferring the patient to the ICU. The differential diagnosis included pheochromocytoma, thyrotoxicosis, and pre-existing viral or peripartum cardiomyopathy. Particularly, pheochromocytoma was suspected on the basis of the disproportionate hyperdynamic response to both ephedrine and direct palpation of the right upper abdominal quadrant. (Ephedrine is an indirect acting vasopressor that stimulates the release of endogenous catecholamines which makes it relatively contraindicated in pheochromocytoma due to potential for massive release of tumoral catecholamines and subsequent hemodynamic instability. Similarly, direct mechanical mobilization (e.g., palpation) of the tumor may result in an extreme hyperdynamic response from a similar (i.e., tumoral catecholamine release) mechanism). On further investigation, a contrasted computed tomography scan revealed a 5 x 4 x 4 cm right adrenal gland mass and the diagnosis of pheochromocytoma was subsequently confirmed with urine metanephrine testing. Later in the year, after optimization by a multidisciplinary team, this patient underwent laparoscopic right adrenalectomy uneventfully. At the time of this writing, both patient and infant are in good health and thriving.

## Discussion

Coexistence of pregnancy and pheochromocytoma is rare. Only 1/5 of cases are identified in the peripartum period, typically as a result of complications 3. In our case, the pheochromocytoma manifested with the onset of abdominal pain/labor, and the diagnosis was facilitated by the use of POCUS. 

Heart disease is a major cause of maternal morbidity and mortality 2, 4. While obstetric critical illness frequently presents on the background of good health and significant physiologic reserve, the obstetric population is evolving to include advanced maternal age, morbid obesity, and heart disease 1. With this reality, obstetric guidelines now emphasize the importance of the skill set to diagnose and manage heart failure in pregnancy, with emphasis placed on early identification of patients who require intervention 2, 4. Dyspnea, a common symptom in the peripartum period, can be benign and self-resolving (e.g., atelectasis, abdominal pain) or an early warning of critical illness (e.g., pulmonary embolism, hemorrhage, heart failure). In this setting, POCUS is an invaluable tool that allows clinicians to narrow down a long list of potential diagnoses (and, importantly, to distinguish between cardiac and respiratory etiologies), facilitating early management before frank clinical decompensation 1, 5, 6. POCUS consists of a guideline-recommended diagnostic modality which allows the treating physician to not only serially assess an unstable patient, but also to guide the response to administered therapies. Specifically, transthoracic echocardiography-guided management of hemodynamic instability adheres to the highest level of evidence according to the American and European societies governing the management of heart failure 5, 7.

Parturients are well-suited to POCUS examination. The lateral and anterior displacement of the heart by the gravid uterus, and the known benefits of aortocaval compression relief in the left lateral position may facilitate image acquisition (except for subcostal views). Indeed, Jain and colleagues found that 93% of term parturients examined with echocardiography had image quality rated as “good” or “perfect” in both the parasternal and apical views. In addition, body mass index (BMI) did not significantly change the quality of images obtained 8. 

Dennis and colleagues published the rapid obstetric screening echocardiography (ROSE) protocol to guide anesthesiologists in assessing cardiac function as the etiology of dyspnea or hemodynamic instability, and it serves as a pragmatic tool guiding cardiac assessment of parturients 1. The ROSE protocol can be supplemented with rapid bedside lung US in emergency (BLUE) protocol^9^ to enhance assessment of peripartum dyspnea. The BLUE protocol provides valuable information about pulmonary pathology^9^ and is, therefore, of importance during pregnancy in which dyspnea is a common complaint and avoidance of ionizing radiation is desirable. Specifically, the analysis of three major lung ultrasound “signatures” (A-lines, B-lines and lung-sliding) offers superior sensitivity (97%) and specificity (95%) in the diagnosis of pulmonary edema 9, 10. Our patient presented a B-line profile that corroborated the diagnosis of cardiogenic dyspnea^11^, ^12^ thereby directing our decision-making to include non-invasive positive pressure ventilation, inotropy and diuresis as part of the treatment.

While several anesthesia residency programs require POCUS training, sufficient infrastructure is not yet ubiquitous to support the role of this technology in our field. Beyond maintaining competency with bedside practice and workshop attendance 13, it is important to have secure digital archives of scans from actual cases to review amongst the clinician group in a systematic fashion for educational purposes 14. Finally, it is important to recruit parturients into research studies^15^ using emerging modalities such as POCUS to develop this body of literature, thereby allowing this group of patients to benefit from ongoing technical advances and knowledge. 

Anesthesiologists play a central role in the management of critically ill obstetric patients, and clinicians skilled in POCUS are able to quickly and accurately diagnose life-threatening pathology and adjust management accordingly. Indeed, the current case serves as a practical example of how POCUS can influence the decision-making and directly (and positively) affect clinical management in a critically ill parturient. 

## Declaration of patient consent 

The authors certify that they have obtained all appropriate patient consent forms. In the form the patient has given her consent for her images and other clinical information to be reported in the journal. The patient understands that her name and initials will not be published and due efforts will be made to conceal her identity, but anonymity cannot be guaranteed.

## Conflicts of Interest

None declared.

## Supplementary Material

Video S1 Parasternal long axis view, showing a dilated and severely hypokinetic left ventricle.

Video S2 Parasternal long axis view with colour Doppler, revealing significant mitral regurgitation.

Video S3 Parasternal short axis view at the mid-papillary muscle level, demonstrating severe left ventricular systolic dysfunction at this level.

Video S4 Apical 4-chamber view, showing a severely dilated left ventricle with severe systolic dysfunction at the basal and mid-levels, along with apical hyperkinesis – referred to as a reverse-Takotsubo cardiomyopathy pattern. Also of note in this context, the right ventricle is hyperkinetic, with no evidence of pressure overload.

Video S5 Apical 4-chamber view, with colour Doppler revealing significant mitral regurgitation.

Graphical Abstract